# Predicting Pathological Complete Response in Rectal Cancer: External Validation of Clinical Models

**DOI:** 10.3390/cancers18152390

**Published:** 2026-07-24

**Authors:** Jesús Pedro Paredes Cotoré, Fernando Fernández López

**Affiliations:** Department of Surgery, University Hospital, School of Medicine, University of Santiago de Compostela (USC)/Complexo Hospitalario Universitario de Santiago de Compostela (CHUS), 15706 Santiago de Compostela, Spain; fernando.fernandez@usc.es

**Keywords:** rectal cancer, pathological complete response, neoadjuvant therapy, prediction model, external validation, organ preservation

## Abstract

**Introduction:** A pathological complete response (pCR) to neoadjuvant therapy is what makes organ preservation possible in rectal cancer, so a reliable pre-treatment estimate of the likelihood of pCR would influence management. Prediction research has focused on imaging and artificial intelligence, leaving the clinical models that any unit should be able to apply largely untested beyond their original cohorts. We validated two such models eiyh our own patients, examined whether local refitting helped, and measured how often pCR occurs across the regimens we use. **Methods:** This was a single-centre cohort of consecutive rectal cancer patients treated with neoadjuvant therapy and resection between 2016 and 2025, drawn from a prospectively maintained registry. pCR was defined as ypT0N0 (no residual invasive tumour in the rectal wall or in the regional lymph nodes on the resection specimen). Two published clinical models (Wang and colleagues; Tan and colleagues) were applied using their original coefficients and assessed for discrimination and calibration; the better-performing model was then adjusted to the local data and its clinical utility was examined via decision-curve analysis. A local six-predictor model was developed and internally validated via bootstrappaing. Reporting followed TRIPOD and STROBE guidelines. **Results:** Of 425 treated and resected patients, 85 achieved pCR (20.0%). The pCR yield differed markedly by regimen, from 27.9% with total neoadjuvant therapy and 22.8% with chemoradiotherapy to 10.3% with short-course radiotherapy. The model of Wang and colleagues separated responders from non-responders only modestly (c-statistic 0.62, 95% confidence interval 0.54–0.69), and its predictors’ effects proved nearly twice as strong in our patients (slope 0.57); the model of Tan and colleagues performed worse (c-statistic 0.58). A locally refitted model performed no better (optimism-corrected c-statistic 0.60). Adjusting the best model to the local data brought its risk estimates closer to observed responses but did not sharpen discrimination, and decision-curve analysis showed only a small net benefit over default strategies. **Conclusions:** Two established clinical models for pCR could only be modestly applied to a contemporary European cohort; the risks they predicted overshot what we observed, and local refitting changed nothing—a low ceiling that lies in the predictors, not their coefficients. A clinical model alone should not be used to identify who is offered organ preservation.

## Simple Summary

After radiotherapy or chemoradiotherapy before surgery, a minority of rectal cancers disappear completely, leaving no tumour in the specimen removed at operation. Patients whose tumour vanishes in these cases tend to have good outcomes, and some can even avoid major surgery and keep their rectum under close surveillance. Being able to predict, before treatment starts, who will respond so well would help doctors and patients plan accordingly. Several simple scoring tools built from routine clinical information have been proposed, but they are rarely tested outside the hospital that created them. We applied two such tools to 425 of our own patients treated over ten years and also rebuilt one using our own data. Neither predicted complete response reliably, and rebuilding it locally did not help; the routine information these tools use was simply not enough. The one factor that clearly influenced complete response was the treatment delivered, not any feature of the patient. Our findings caution against using these clinical scores alone to decide who may safely keep their rectum and support continued reliance on post-treatment assessment with imaging and endoscopy.

## 1. Introduction

Colorectal cancer is the third most commonly diagnosed cancer and the second leading cause of cancer death worldwide, with a substantial proportion arising in the rectum [1]. For locally advanced disease, neoadjuvant treatment before total mesorectal excision has been the standard for two decades: preoperative chemoradiotherapy improves local control over a postoperative schedule and does so with less toxicity [2], and adding fluoropyrimidine chemotherapy to radiotherapy improves local control further [3]. More recently, total neoadjuvant therapy, which involves delivering the systemic component before surgery, has raised both disease-free survival and the proportion of tumours that respond completely [4,5]. Between 15% and 27% of rectal cancers treated in this way show no residual tumour at pathology, yielding a pathological complete response (pCR), the one outcome of neoadjuvant treatment that approaches cure without resection; these patients exhibit markedly lower recurrence and better survival [6]. Patients who sustain a clinical complete response can now be offered organ preservation, with radical surgery deferred unless the tumour regrows [7,8,9], and total neoadjuvant therapy has been used explicitly to improve this opportunity [10]. Identifying such patients before treatment begins is therefore of real clinical value.

The prediction literature has moved away from the clinic. Effort has been focused on radiomics and deep learning derived from magnetic resonance imaging, where external areas under the receiver operating characteristic curve—a summary measure of how well a model separates responders from non-responders, ranging from 0.5 (no better than chance) to 1.0 (perfect)—above 0.8 are now common, reported alongside methods that vary between studies and seldom incorporate routine clinical data [11]. A model built only from what a tumour board already records—clinical stage, carcinoembryonic antigen, histological type, and the selected regimen—needs neither dedicated software nor a radiologist, yet has almost never been tested beyond the centre that developed it [12,13,14]. Two questions determine whether it belongs in practice: does it retain its discrimination in a population that did not develop it, and, where it underperforms, does local refitting recover any lost performance?

Trial pCR rates overstate everyday practice; once unselected patients and de-escalated schedules are included, the proportion falls, and it tracks the regimen more closely than any feature of the patient [15]. At a Spanish tertiary-care centre, over a consecutive decade of practice, we addressed four questions: how often pCR occurs across the regimens we deliver, whether the models of Wang and of Tan transport on their original coefficients, whether refitting those predictors locally recovers performance, and whether the best of them offers clinical utility on decision-curve analysis. Reporting follows the TRIPOD and STROBE statements [16,17].

## 2. Materials and Methods

### 2.1. Study Design and Setting

This was a retrospective analysis of a prospectively maintained single-centre rectal cancer registry at one tertiary teaching hospital, covering patients treated over a consecutive decade (2016–2025). The registry records consecutive patients—that is, every eligible patient managed in the period, enrolled without selection, which limits selection bias at the point of cohort assembly—with histologically confirmed rectal adenocarcinoma discussed at the institutional colorectal multidisciplinary meeting, as well as their staging, treatment, pathology, and follow-up. The present work was specified as an external validation of published pre-treatment models for pCR, supplemented by a local development model; the prediction questions and the predictor set were fixed before the validation analyses were carried out. Because the cohort was assembled for clinical care rather than for this question, the analysis was observational and post hoc by construction, and the resulting selection is acknowledged as a limitation. Reporting follows the TRIPOD statement for prediction model studies and the STROBE statement for observational studies [16,17].

### 2.2. Participants and Staging

**Figure 1:**
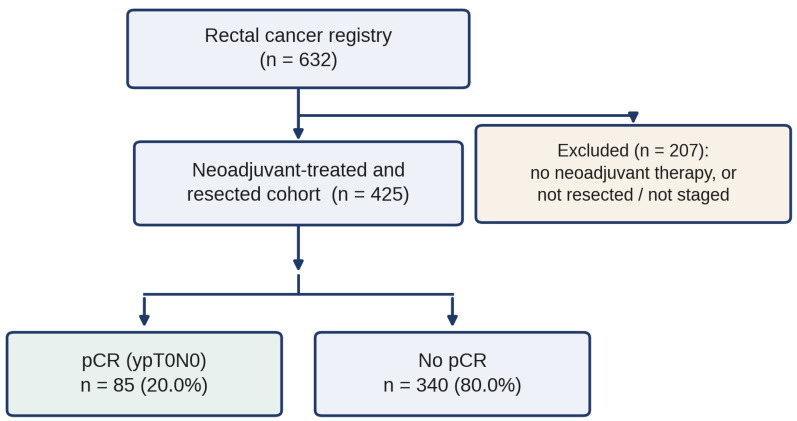
Flow of patients through the study, from the rectal cancer registry to the analytic cohort of neoadjuvant-treated and resected patients with assessable pathological response.

Eligible patients had histologically proven rectal adenocarcinoma, received neoadjuvant therapy, and subsequently underwent resection with pathological assessment of the specimen. The diagnosis of adenocarcinoma was established by routine diagnostic histopathology (haematoxylin and eosin) as part of standard clinical care; this study is registry-based and did not include immunohistochemical or molecular analyses. Patients managed without resection (including those who entered a watch-and-wait pathway after a clinical complete response) and those whose specimen could not be staged were not analysable for the outcome and were excluded (Figure 1); the local model was therefore developed among operated-on patients only. Baseline staging followed institutional protocol and national guidance, with pelvic magnetic resonance imaging for local staging and thoraco-abdominal computed tomography for distant disease; magnetic resonance interpretation and reporting followed the European Society of Gastrointestinal and Abdominal Radiology consensus [18], and treatment decisions followed European Society for Medical Oncology guidance current at the time [19]. Clinical stage was recorded using the TNM system in force during the study period. Patients were not selected on the basis of tumour height, age, or comorbidity beyond eligibility for neoadjuvant therapy and resection, so the cohort reflects the unselected case-mix referred to as a single unit across the period.

### 2.3. Neoadjuvant Treatment and Surgery

Neoadjuvant treatment was allocated by the multidisciplinary team according to tumour stage, height, and patient fitness and fell into four categories that are reported separately. Long-course chemoradiotherapy administered approximately 45 to 50.4 Gy in 25 to 28 daily fractions with a concurrent fluoropyrimidine, the schedule established by randomised trials as standard for locally advanced disease [2,3,20]. Short-course radiotherapy administered 25 Gy in five fractions, given either immediately before surgery or, in more recent years, followed by consolidation chemotherapy in a total-neoadjuvant configuration [21]. Total neoadjuvant therapy combined a full course of induction or consolidation chemotherapy with chemoradiotherapy before surgery [4,5]. A small group received radiotherapy alone (radiotherapy without any concurrent or sequential systemic agent), usually because comorbidity precluded chemotherapy. Resection involved total mesorectal excision, undertaken after an interval from the end of radiotherapy that was typically eight to twelve weeks, and in keeping with randomised evidence, it was not deliberately prolonged in order to increase response [22]. The choice and completeness of each regimen were taken from the treatment record. Adjuvant chemotherapy after surgery was administered according to the pathological stage and multidisciplinary decision and did not form part of the pre-treatment prediction question.

### 2.4. Outcome

The primary outcome was pCR, defined as the absence of residual tumour in the primary and in all examined regional lymph nodes (ypT0N0) on the resection specimen [6]. Tumour regression was additionally graded on the five-point Mandard scale, from complete regression to no regression [23]; a major pathological response was defined as the two best grades, and T-downstaging as a lower pathological response than the clinical T category. These secondary descriptors were examined for context only, since regression grade carries independent prognostic weight after chemoradiotherapy [24]. A pathological assessment was performed by gastrointestinal pathologists as part of routine care and was, by nature, performed without reference to the predictor values, so outcome ascertainment was blind to the models being tested.

### 2.5. Predictors and Published Models

Two published clinical models were selected in advance based on the criterion that every predictor was computable from the registry, excluding models that required radiomic features, neoadjuvant haemoglobin levels, or the neutrophil-to-lymphocyte ratio. Several other pre-treatment models for pCR have been published, including image-based radiomic and deep learning models [11] and other clinical nomograms [12]; these were not eligible for the present validation because they require inputs that a routine clinical registry does not hold, such as radiomic- or magnetic resonance-derived features or laboratory-derived ratios. The models of Wang and of Tan were chosen because they are purely clinical and recently published, and every one of their predictors was computable from routinely recorded data, making them the models most likely to be usable in ordinary practice. The model of Wang and colleagues combines pre-treatment carcinoembryonic antigen, histology, clinical T and N stage, magnetic resonance extramural vascular invasion, and the use of total neoadjuvant therapy [13]. The model of Tan and colleagues, derived in a population dataset, uses clinical T and N stage, mucinous histology, and the pre-treatment carcinoembryonic antigen [14]. Each predictor was mapped to its registry variable; the clinical nodal category (N0/N1/N2) was derived from the clinical TNM stage group, and the carcinoembryonic antigen was dichotomised at 5 ng/mL where the source model required it. Where a model specified the transformation of a predictor, that transformation was reproduced exactly.

### 2.6. Statistical Analysis

Analyses followed a prespecified plan structured around the accepted components of prediction model validation: discrimination, calibration and clinical usefulness [25]. The pCR rate is reported overall and by neoadjuvant regimen with 95% confidence intervals. For each published model, the linear predictor was computed from the original published coefficients without refitting; discrimination was summarised by the concordance (c) statistic with bootstrap confidence intervals, and the calibration slope was obtained by regressing the observed outcome on the linear predictor, with a slope below one indicating that the published effects are too strong for this population [26]. Neither source publication reported the model intercept, so the original absolute calibration (calibration-in-the-large) could not be tested; for the better-discriminating model, intercept and slope were re-estimated in-sample (recalibration), and clinical utility was examined by decision-curve analysis across a plausible range of decision thresholds, compared with the default strategies of offering organ preservation to all or to none [27]. A model is clinically useful only where its net benefit exceeds both defaults across the threshold probabilities at which a clinician would actually act; for an organ preservation decision, this threshold encodes how many unnecessary avoidances of surgery one would accept to avoid missing a single true responder, and we regard a net benefit that is both small and confined to a narrow band of thresholds as clinically unpersuasive. A local model, developed in our own institution using the study cohort, with the same six pre-treatment predictors, was fitted by logistic regression and internally validated by bootstrap optimism correction over 1000 replications; with 73 events among the predictors modelled, the events-per-variable ratio was 12.2, which is above the conventional minimum. Head-to-head comparisons used identical patients, with the number of patients and events stated. The full cohort of 425 patients was used for the descriptive analyses (the pCR rate, regimen-specific rates and regression grade). The model analyses required every predictor to be present: 348 of 425 patients (82%) had complete data, and the remaining 77 were excluded from those analyses because at least one predictor was missing. Missingness was concentrated in magnetic resonance extramural vascular invasion and, less often, in pre-treatment carcinoembryonic antigen. We used complete-case analysis and did not perform multiple imputation, because the excluded patients closely resembled those retained (similar age, clinical T stage, and carcinoembryonic antigen, and a comparable pCR rate, 16% versus 21%); imputation would therefore have added modelling assumptions without materially changing the estimates. Regimens were not formally compared with each other for pCR; since this study was not powered for such comparisons, regimen-specific rates are only descriptive. This manuscript was prepared in line with the TRIPOD explanation-and-elaboration guidance, and the study was appraised against the risk-of-bias and applicability domains of PROBAST [28,29]. Confidence intervals for the c-statistic were obtained from 1000 bootstrap resamples, and the same resampling underpinned the optimism correction of the locally developed model. The two imported models were compared with the local model on an identical set of patients with complete predictor data, so no difference in discrimination could arise from differences in case-mix. No adjustment for multiplicity was made, since the analyses were few and prespecified and the study is explicitly descriptive rather than confirmatory. Statistical analyses were performed in Python (version 3.11) using the NumPy (version 3.11) and pandas libraries; the logistic models, bootstrap resampling, and decision-curve analysis methods were implemented directly in code. During preparation, a generative artificial intelligence assistant (Claude, Anthropic) supported language editing; all data, analyses, and interpretations were verified and approved by the authors, who take full responsibility for the content.

## 3. Results

### 3.1. Cohort and Real-World Response

The analytic cohort comprised 425 patients treated with neoadjuvant therapy and resected (Figure 1); baseline characteristics by response status are shown in Table 1; the two response groups were broadly similar at baseline, with no material difference in age or sex and a clinical stage distribution reflecting the locally advanced case-mix for which neoadjuvant therapy is indicated. pCR was reached in 85 patients (20.0%, 95% confidence interval 16.5–24.1), yielding a figure consistent with real-world series rather than trial series [15]. The response factor tracked the intensity of the regimen: pCR occurred in 27.9% of patients administered total neoadjuvant therapy and 22.8% of those administered long-course chemoradiotherapy, but in only 10.3% after short-course radiotherapy and 3.8% after radiotherapy alone—that is, radiotherapy delivered without concurrent chemotherapy (Table 2). A major pathological response was recorded in 31.2% of the cohort, and T-downstaging in 263 of 407 patients with paired clinical and pathological staging (64.6%).

### 3.2. External Validation of Published Models

**Figure 2:**
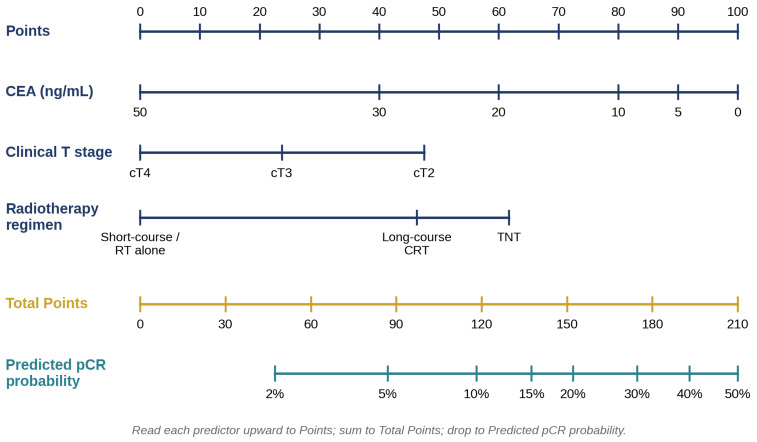
Receiver operating characteristic (ROC) curves for the prediction of a pathological complete response (pCR) by the imported model of Wang and colleagues, the imported model of Tan and colleagues, and the locally refitted model, all computed on an identical set of 348 patients with complete predictor data. Each curve plot’s sensitivity was measured against avalue of 1—specificity across all probability thresholds; the diagonal denotes discrimination no better than chance (area under the curve 0.5), and trajectories approaching the upper-left corner indicate better discrimination. The areas under the curve were 0.62 for the model of Wang and colleagues, 0.58 for that of Tan and colleagues, and 0.60 (optimism-corrected) for the local model, indicating modest discrimination for all three.

External validation measures a model against the performance originally reported for it, so the comparisons below are descriptive rather than interpretive. With its original coefficients, the model of Wang and colleagues discriminated modestly for pCR (c-statistic 0.62, 95% confidence interval 0.54–0.69; 348 patients, 73 events), well below the 0.78 reported in its own external validation cohort [13]. Its calibration slope was 0.57, indicating that the published predictor effects are roughly half as strong in this population. The model of Tan and colleagues discriminated less well (c-statistic 0.58, 95% confidence interval 0.51–0.65; calibration slope 0.87) (Table 3; Figure 2).

### 3.3. Local Refitting and Clinical Utility

**Figure 3:**
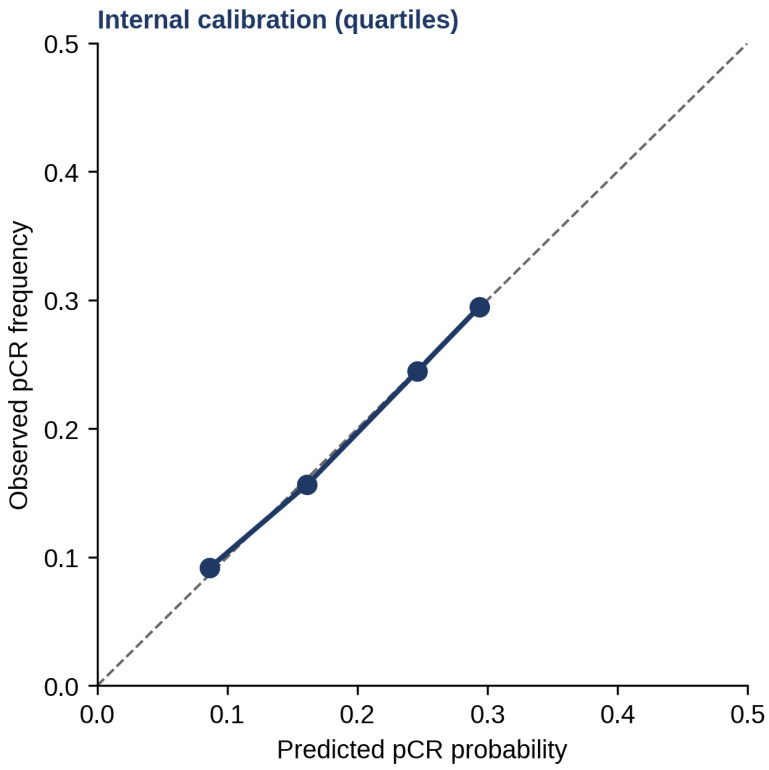
Calibration of the model of Wang and colleagues after in-sample recalibration, showing predicted versus observed pathological complete response by quartile of predicted probability. Decision-curve analysis of the recalibrated and local models compared with the strategies of offering organ preservation to all patients or to none.

Refitting the same six predictors to the local data did not recover performance: the developed model reached an apparent c-statistic of 0.63 and an optimism-corrected c-statistic of 0.60, which is statistically indistinguishable from the imported model of Wang and colleagues on the same 348 patients (0.63 versus 0.62; Tan 0.58). Recalibrating the model of Wang and colleagues (intercept −1.35, slope 0.57) brought predicted and observed pCR into agreement but, as expected, left discrimination untouched at 0.62; the recalibrated probabilities spanned only about 12% to 31%, so the model separates responders from non-responders weakly even when correctly centred (Figure 3). On decision-curve analysis, the recalibrated and local models yielded a small positive net benefit over offering organ preservation to all or to none across thresholds of roughly 0.20 to 0.35, but the margin was narrow and disappeared at lower thresholds (Figure 3).

### 3.4. Response by Regimen and Clinical Stage

Examining response by regimen within nodal strata revealed a counter-intuitive pattern: clinically node-negative tumours achieved pCR less often than node-positive ones (15% versus 22% overall), which is a reversal of the usual direction. The difference was partly a matter of treatment selection—node-negative tumours were more often given short-course radiotherapy, the regimen with the lowest yield—but it persisted within the chemoradiotherapy subgroup, where node-negative tumours achieved pCR in 7 of 40 (17.5%; 95% confidence interval 8.7–32.0) and node-positive tumours in 55 of 210 (26.2%; 95% confidence interval 20.7–32.5) (Table 4), so it is not entirely explained by regimen. These subgroups are small, and their confidence intervals overlap widely; the observation is exploratory and hypothesis-generating.

## 4. Discussion

Two published clinical models were applied to an independent European cohort, and neither transferred well. The stronger one, Wang’s, fell from the 0.78 of its development series to 0.62 in this cohort, with its coefficients describing effects nearly twice as strong as those we could measure (calibration slope 0.57). The refit is decisive: rebuilt on our own 425 patients, the same six variables reached the same modest ceiling, with nearly identical apparent and optimism-corrected estimates. Whether the model is imported or rebuilt, the result is unchanged, which locates the limitation in the predictors rather than in their coefficients. Cohort heterogeneity and treatment selection effects are real, and we discuss them below, but they cannot be the whole story: had discrimination been held back chiefly by case-mix or by coefficients ill suited to our population, refitting on our own data would have lifted it—and it did not. External discrimination can fall for several reasons—a different case-mix or outcome prevalence, predictor–outcome associations that differ between settings, differences in how predictors are measured, or predictors that are simply weak—and the refit isolates the last of these, since re-estimating the coefficients in the validation data leaves discrimination unchanged only when the predictors themselves carry little signal. Clinical stage, a single tumour marker, and a treatment category do not carry enough information for identifying which tumour will achieve pCR.

This limitation explains the field’s turn to imaging. Radiomic and deep learning models have reported better discrimination in the literature, plausibly because diffusion, texture, and interval changes capture information that a TNM category and a single carcinoembryonic antigen value cannot [11]; we could not test this directly, since such features were not held in our registry. None of this discredits the clinical model; it defines its proper role. These variables rank a population’s baseline risk adequately but should not certify an individual patient as safe for watchful waiting, and the response assessment that guides organ preservation is itself imaging-led, structured around restaging magnetic resonance imaging and endoscopy [18]. Recalibration sharpened rather than softened this limitation: re-centring confined the predicted probabilities to roughly 12 to 31%, a range that identifies no patient as a high-probability responder, and decision-curve analysis showed net benefit over the default strategies only across a narrow band of thresholds. In practical terms, this means the model could not influence a management decision on its own: a predicted probability that never rises clearly above about one in three does not identify a patient in whom organ preservation could be offered—or in whom radical resection could be safely withheld—with any confidence, so the decision continues to rest on post-treatment response assessment.

The regimen gradient warrants emphasis. Complete response fell from about a quarter of patients after total neoadjuvant therapy or chemoradiotherapy to roughly one in ten after short-course radiotherapy—the regimen, more than tumour biology, driving the difference [15]. This ordering matches the randomised evidence, in which intensified and total-neoadjuvant schedules produce higher complete-response rates than radiotherapy-based de-escalation, built on a chemoradiotherapy backbone whose local control benefit is durable at ten years [4,5,21,30]. The nodal data extend this caution: clinically node-negative tumours, those more often allocated to de-escalated radiotherapy, achieved pCR less often, and the gap persisted within the chemoradiotherapy subgroup, where treatment was held constant. The subgroups are too small to support a firm conclusion, but they are large enough to illustrate that an apparent predictor of response may instead mark the treatment a patient was selected to receive. Two mechanisms plausibly underlie the inverse association: clinical nodal status is assigned by imaging and is an imperfect measure, and node-positive tumours were preferentially allocated to the more intensive, higher-yield schedules. The within-regimen stratification in Table 4 acts as a sensitivity analysis, holding treatment constant; a formal multivariable adjustment was precluded by the small size of the resulting subgroups.

The limitations are substantial and worth stating precisely. This is a single-centre, retrospective registry assembled for clinical care rather than for answering questions; selection is intrinsic to the data and cannot be excluded. Neither Wang nor Tan published an intercept, which allowed the assessment of discrimination, the calibration slope, and a local re-centring but not the models’ original absolute accuracy; we state this rather than allow the recalibrated curve stand in for it. The clinical nodal category was derived from the stage group rather than recorded separately, mucinous tumours were too few to analyse, and the event count, adequate for the analyses performed, left the confidence intervals wide. The local model was restricted to the same six predictors deliberately, to separate weak coefficients from weak predictors: with the univariable signal concentrated almost wholly on the carcinoembryonic antigen, clinical T stage, and regimen. And with only 73 events available, a longer candidate set would have risked overfitting without raising the ceiling. By design and by data availability, we validated only clinical models: radiomic, deep learning, molecular, and circulating-biomarker models—which the literature suggests predict pCR better—require inputs that a routine registry does not hold, so a head-to-head comparison against them in this cohort was not possible, and their absence is a limitation of this study’s scope. Among routinely available variables not modelled separately here, extramural vascular invasion is already a component of one of the validated models, while tumour height (distance from the anal verge), circumferential resection margin involvement, body mass index, additional magnetic resonance features, and, where available, molecular markers are plausibly informative and would be worth testing in larger series with more events. pCR is a surrogate for outcomes not yet measurable here; follow-up in this cohort is too short for survival analysis, and we relied on the established pCR–survival association rather than re-establish it [6]. The stakes of correct selection are nonetheless high, since watch-and-wait is oncologically safe only when complete response is correctly identified and most local regrowths are salvageable under close surveillance [31]. Even allowing for these limitations, the clinical message is firm: a clinical model cannot, by itself, select a patient for organ preservation, and the assessment that can—at present, an imaging-led one—remains necessary.

For real-world practice, these findings argue against using a routine clinical model as a gatekeeper for organ preservation and favour reserving it for what it does adequately—describing the baseline probability of the response for a group, informing consent, and helping set expectations before treatment begins. Two developments could shift the balance. Combining routine clinical variables with MRI radiomics, deep learning, circulating tumour DNA, inflammatory and immune markers, and explainable machine-learning methods—in integrated, multimodal frameworks—may lift discrimination past the ceiling seen here, and prospective multicentre registries with standardised response assessments would allow such combined models to be validated properly rather than developed and tested on the same data. Until then, the defensible position is that no model built on the stage, a single marker, and the regimen should be asked to make an organ preservation decision on its own, and that external validation—not development performance—is the only fair test of whether any model, clinical or imaging-based, is ready for the clinic [25,29].

## 5. Conclusions

These findings come from a single centre, retrospective design and a cohort in which the discrimination we could measure was modest, and they should be read in that light. Within these limits, two established clinical models for pathological complete response could be only modestly transferred to a contemporary European cohort; the risks they predict overshot what we observed, and rebuilding the same predictors locally did not raise the ceiling—a limit that lies in the routinely available predictors themselves rather than in their coefficients. The strongest and most reproducible signal was not a patient feature but the treatment delivered: complete response was far more frequent after chemoradiotherapy or total neoadjuvant therapy than after de-escalated schedules. A clinical model built on the stage, a single tumour marker, and the treatment category can rank a population’s baseline probability of response but cannot, on its own, certify an individual patient as safe for organ preservation; such a decision still requires the post-treatment clinical and imaging-based assessment of patient’s response, to which such a model is a complement at best.

## Figures and Tables

**Table 1:** Baseline characteristics by pathological complete response status.

Characteristic	pCR (n = 85)	No pCR (n = 340)	*p*
Age, years	67.0 (61.0–73.0)	69.5 (59.0–77.0)	0.09
Male sex	52 (61%)	221 (65%)	0.60
BMI, kg/m^2^	26.9 (25.0–29.5)	26.6 (24.4–29.6)	0.52
CEA, ng/mL	1.6 (0.8–3.1)	2.4 (1.2–6.0)	0.002
Tumour height, cm	7.0 (5.0–10.0)	8.0 (5.0–10.0)	0.20
cT2	3 (4%)	4 (1%)	
cT3	63 (74%)	226 (66%)	
cT4	14 (16%)	96 (28%)	
cN positive	70 (82%)	242 (71%)	0.05
EMVI positive (MRI)	16 (19%)	89 (26%)	0.21
Mucinous/signet histology	4 (5%)	19 (6%)	0.96
Total neoadjuvant therapy	12 (14%)	31 (9%)	

Values are median (interquartile range) or n (%). pCR, pathological complete response (ypT0N0); BMI, body mass index; CEA, carcinoembryonic antigen; EMVI, extramural vascular invasion; MRI, magnetic resonance imaging. p from Mann–Whitney U or χ^2^ test; cT categories are descriptive.

**Table 2:** Pathological response by neoadjuvant regimen and secondary response endpoints.

Regimen/Endpoint	n	pCR, n (%) [95% CI]
Total neoadjuvant therapy	43	12 (27.9) [16.7–42.7]
Long-course chemoradiotherapy	285	65 (22.8) [18.3–28.0]
Short-course radiotherapy	68	7 (10.3) [5.1–19.8]
Radiotherapy alone (long)	26	1 (3.8) [0.7–18.9]
Major pathological response (TRG 1–2)	425	132 (31.2)
T-downstaging (ypT < cT)	407	263 (64.6)

pCR, pathological complete response; CI, confidence interval; TRG, tumour regression grade. Denominators differ by data availability.

**Table 3:** External validation and head-to-head discrimination for pathological complete response.

Model	n	Events	c-Statistic (95% CI)	Calib. Slope
Wang et al. [13] (imported)	348	73	0.62 (0.54–0.69)	0.57
Tan et al. [14] (imported)	366	77	0.58 (0.51–0.65)	0.87
Local model (refitted)	348	73	0.60 (corrected)	

Head-to-head on identical patients (n = 348, 73 events): Wang 0.62, Tan 0.58, local 0.63 (apparent). The local model c-statistic is optimism-corrected by bootstrap. Published model intercepts were not available, so original absolute calibration could not be tested. CI, confidence interval.

**Table 4:** Pathological complete response by regimen within clinical nodal strata.

Regimen	cN0, pCR/n (%)	cN+, pCR/n (%)
Long-course chemoradiotherapy	7/40 (18%)	55/210 (26%)
Total neoadjuvant therapy	1/3 (33%)	11/39 (28%)
Short-course radiotherapy	3/24 (12%)	4/41 (10%)

pCR, pathological complete response; cN0, clinically node-negative; cN+, clinically node-positive. Subgroup numbers are small and the comparison is hypothesis-generating.

## Data Availability

The data that support the findings of this study are not publicly available owing to patient-privacy constraints but are available from the corresponding author upon reasonable request, subject to institutional approval.
